# A Social Network Analysis of Social Cohesion in a Constructed Pride: Implications for Ex Situ Reintroduction of the African Lion (*Panthera leo*)

**DOI:** 10.1371/journal.pone.0082541

**Published:** 2013-12-20

**Authors:** Jackie Abell, Morgan W. B. Kirzinger, Yvonne Gordon, Jacqui Kirk, Rae Kokeŝ, Kirsty Lynas, Bob Mandinyenya, David Youldon

**Affiliations:** 1 Coventry University, Coventry, United Kingdom; 2 African Lion & Environmental Research Trust, Livingstone, Zambia; 3 University of Regina, Regina, Saskatchewan, Canada; 4 African Lion & Environmental Research Trust, Antelope Park, Gweru, Zimbabwe; 5 African Lion & Environmental Research Trust, Livingstone, Zambia; 6 African Lion & Environmental Research Trust, Livingstone, Zambia; 7 African Lion & Environmental Research Trust, Antelope Park, Gweru, Zimbabwe; 8 African Lion & Environmental Research Trust, Antelope Park, Gweru, Zimbabwe; 9 African Lion & Environmental Research Trust, Livingstone, Zambia; Semmelweis University, Hungary

## Abstract

Animal conservation practices include the grouping of captive related and unrelated individuals to form a social structure which is characteristic of that species in the wild. In response to the rapid decline of wild African lion (*Panthera leo*) populations, an array of conservational strategies have been adopted. Ex situ reintroduction of the African lion requires the construction of socially cohesive pride structures prior to wild release. This pilot study adopted a social network theory approach to quantitatively assess a captive pride’s social structure and the relationships between individuals within them. Group composition (who is present in a group) and social interaction data (social licking, greeting, play) was observed and recorded to assess social cohesion within a released semi-wild pride. UCINET and SOCPROG software was utilised to represent and analyse these social networks. Results indicate that the pride is socially cohesive, does not exhibit random associations, and the role of socially influential keystone individuals is important for maintaining social bondedness within a lion pride. These results are potentially informative for the structure of lion prides, in captivity and in the wild, and could have implications for captive and wild-founder reintroductions.

## Introduction

The IUCN classifies the African lion as ‘vulnerable’. The speed of decline in wild populations as a consequence of increases in human population, fragmentation of habitat, human-wildlife conflict (HWC), climate change, inbreeding depressions, and disease, have led to the emergence of an array of in situ and ex situ strategies to conserve the species. These include reintroduction [1], [2]. A recent estimate indicates 32,000–35,000 wild lions are left in the wild [3]. With an estimated 30% decline in two decades [4] it has been argued that wild populations may now need to be supplemented and restored from captive-bred founders [5], [6]. However, reintroductions are hampered by poor success rates, especially those which use captive-bred founders [7], [8], [9]. Even where wild-founders are used, the failure to socially bond introduced lions with existing pride members has resulted in a failed reintroduction [1], [2]. Consequently, where the target species for ex situ reintroduction is social, efforts must ensure cohesive group structures are in place before wild release is attempted [10]. The increasing need for animal conservation has led to practices where related and unrelated individuals live together in groups either in a zoo, a game reserve, or as part of a reintroduction programme [11].

Unfortunately, establishing social cohesion between group members in captive-bred animals has been considered problematic owing to the environment in which the species is housed and their relationships with humans [12], [13]. Moreover, there is scant empirical study on group cohesion in social animals per se. That said, successful wild releases of captive-bred lions have occurred and have highlighted the importance of the creation of a pride structure to ensure their survival [14], [15]. This paper examines social cohesion within a constructed pilot pride of captive-bred lions, released into a fenced environment, free from human contact. The pride exists as part of an ex situ conservation programme which has established criteria for success for this part of the programme as being that released prides are socially cohesive and self-sustaining. Once these criteria have been met, the pride is moved to a larger managed ecosystem (+10,000ha) containing competitors (e.g. hyena) as well as a broader range of prey species. Cubs born into these prides are candidates for release into the wild having received no human interaction or interference.

Lions are the most social of the Felidae species [16] residing in prides characterised as fission-fusion societies. Such social organizations are perhaps better defined as fission-fusion dynamics, as they vary across and within social species in terms of how cohesive their members are [17]. Lion prides are typically highly cohesive and it is proposed that a pride of lions exhibits the “*evolutionary crucible in which a cooperative society is formed*” [18]. Social cohesion and cooperation are the cornerstone of lion behaviour, providing the species with significant benefits from living in groups, including defence of a territory to increase the reproductive success of the group [19], coordinated hunting [20] and communal nurturing of young hence increasing survival rates of young [18]. Such cooperation in wild lions is ordinarily based on kinship, as prides comprise of related females. Female lions rarely leave their natal territory, and unrelated females from different prides do not ordinarily join together to form new ones [21]. As such, social cohesion within lion prides lends itself to explanation from Hamilton’s (1964) kinship selection theory, where cooperation is based on degrees of genetic relatedness [22]. Pride members are helpful to each other on the basis of direct and indirect fitness benefits, which take the form of directing advantageous behaviour towards kin (in the form of food sharing, nursing young, defence, and so on) and outbreeding [23].

Studies specifically on social cohesion in wild lion prides are surprisingly scant. One of the most comprehensive studies comes from Schaller’s (1972) study of Serengeti lions [24]. From his field observations, Schaller identifies social behaviours indicative of social cohesion. He reports greeting by head rubbing and social licking are the two behaviours which facilitate group cohesion more than any other. A possible function of play between adult lions might be to strengthen social bonds between them [24]. It is appropriate to note here that the role of play in social mammals has been debated empirically for its evolutionary and functional value [25], [26], [27], [28]. Social interactions have also been documented in other social species for their role in maintaining cohesive bonds between individual members. For example, the role of spatial proximity has been linked to social proximity in animal social networks, and has been studied in released captive-raised chimpanzees (*Pan troglodytes*) to assess relationships between members and overall social structure [29]. Smith et al. (2011) document the use of social greetings between hyenas to facilitate social bonding [30].

Whilst group living provides species such as lions with a social network, interactions within it are not evenly distributed [31], [24]. Interaction patterns in animal or human networks are not random but display individual preferences to associate with particular members. This structure governs interactions between members and as a result the speed and direction at which information, disease, and behavioural strategies are communicated through the network depends on which individuals are in receipt of it [32]. The most connected members of a social network are termed ‘keystone individuals’ and play a critical role in fission-fusion societies, providing overall stability and cohesion [33]. Although common in the social sciences, Social Network Theory (SNT) remains under-used in understanding animal social networks [32], [33], [34]. SNT has proved to be a useful tool for assessing and quantifying social structure at a group and individual level [33], [34], [35], [36]. A structural network contains nodes and edges; nodes denote individual animals that constitute the group and edges signify the strength of the relationship between them. Networks can be created for a range of interactions which may show individuals as centrally connected for some behaviours (e.g. play) but not for others (e.g. greeting). The analysis of a social network utilises a series of metrics to enable direct contact measures including degree (how many social partners each individual has) and indirect measures such as betweenness or centrality (how important an individual is as a point of social connection for all members of the group) and density (the number of connections within a group). At a group level, high values represent greater levels of connectedness across a network than low values. At an individual level, high values denote centrality to the network and relative position vis a vis other individuals within it as a function of interaction type. Social network analysis can also be used to assess associations between attribute characteristics of individuals (such as sex, age, and genetic relatedness) and relationships with other individuals in the network.

The current study concerns social cohesion and bondedness in a constructed lion pride. In accordance with existing work on social cohesion in lions, the study focuses on social licking, greeting, and play to document connectedness within the pride. The documenting of these behaviours and their directionality lends itself to a study of social dominance, although existing research remains mixed about the presence of dominance patterns between adult female lions. Studies of dominance have not typically focused on interaction patterns and the influence alphas have on other individuals in their group as a consequence of their connectedness [33]. With respect to lions, investigations of hierarchical relationships have focused on reproductive behaviour [20]. A social network analysis can facilitate quantitative measures to provide information on the existence of social power, as well as social influence, within a pride [32], [33], [34]. The direction of social behaviours (grooming, licking, play) was noted and were analysed as asymmetric (directional) networks. Social network analysis also facilitates study into group composition, such that we can examine any relationship between spatial proximity and social proximity, as identified in chimpanzee societies [29]. Therefore, this study utilises SNT to examine cohesion within a pilot pride of captive-bred lions, comprising of related and unrelated adults, and their wild-born cubs. It considers the connections between individual lions in relation to specific social behaviours, and the role of kinship in these networks. As there are no current social network analyses upon lion prides, the networks presented here cannot be compared to existing ones. Furthermore, whilst this study is unable to make predictions about social cohesion in general it seeks to offer insights into bondedness from a captive-founded pride. As noted, research on captive big cats can provide information on behaviour which would otherwise be impossible or difficult from their wild counterparts [37].

## Materials and Methods

### Research Ethics

Ethical approval for this work was obtained from the African Lion & Environmental Research Trust ethics committee, who own the lions involved. This work was carried out in strict accordance with ALERT’s husbandry, welfare and ethical protocols for lions, which were developed in accordance with guidelines prepared by the Zoological Society of San Diego, and PAAZAB and meet Zimbabwean legal requirements. Research protocols were created and implemented by ALERT.

### Study Site and Animals

The study was undertaken in a managed wild ecosystem. The ALERT privately owned 163 ha. Ngamo lion release site is situated 13 km outside Gweru in central Zimbabwe. The site comprises of mixed dry miombo woodland and open grassland on undulating topography (ranging 1370–1398 m above sea-level). The Ngamo pride consisted of 12 lions throughout the duration of the study (Jan 6^th^ 2012–29^th^ March 2013). These comprised of one adult male (MI) aged 9 years, 6 adult females (AS, KE, KW, NL, NR, PH) aged between 7–8 years, and 5 cubs of which AT1 was 12 months old, and AS4, AS5, KE3 and KE4 were 2–3 months old when the study began. Genetic kinship relations within this pride are presented in Table 1. The females of the Ngamo pride were released into the site on 1^st^ September 2010, and the adult male (MI) joined them two weeks later.

**Table 1:** Coefficient of genetic relationship between individual lions in the Ngamo pride.

	AS	AS4	AS5	AT1	KE	KE3	KE4	KW	NL	NR	PH
AS		0.5	0.5	0.25	0.25	0.125	0.125	0.25	0	0	0.25
AS4	0.5		0.5	0.313	0.125	0.313	0.313	0.125	0	0	0.125
AS5	0.5	0.5		0.313	0.125	0.313	0.313	0.125	0	0	0.125
AT1	0.25	0.313	0.313		0.125	0.313	0.313	0.125	0	0	0.125
KE	0.25	0.125	0.125	0.125		0.5	0.5	0.5	0	0	0.25
KE3	0.125	0.313	0.313	0.313	0.5		0.5	0.25	0	0	0.125
KE4	0.125	0.313	0.313	0.313	0.5	0.5		0.25	0	0	0.125
KW	0.25	0.125	0.125	0.125	0.5	0.25	0.25		0	0	0.25
NL	0	0	0	0	0	0	0	0		0.5	0
NR	0	0	0	0	0	0	0	0	0.5		0
PH	0.25	0.125	0.125	0.125	0.25	0.125	0.125	0.25	0	0	

NL and NR arrived at ALERT as inbred cubs. Their mother and father were full-siblings. Both lions have been spayed.

The Ngamo release site has naturally occurring prey species including steenbok (*Raphicerus campestris)* and common duiker (*Sylvicapra grimmia*). Impala (*Aepyceros melampus*), plains zebra (*Equus burchelli*) and wildebeest (*Connochaetes taurinus*) were introduced into the site prior to release, and restocked during the period of the study. Introduced game came from predator-aware and non-predator-aware stocks. The site is sufficiently large for prey species to evade predation each and every time they are hunted.

### Data Collection

Data collection was undertaken by trained research technicians as part of their regular monitoring of the pride. Data collected from the Ngamo pride between 6^th^ January 2012–31^st^ March 2013 denotes a period when the pride was in the process of raising their first litters of cubs. An enclosed research vehicle entered the site up to three times a day for the time periods: 6∶30–8∶30; 11∶00–13∶00; 16∶00–18∶00. This resulted in 1352 data-points for group composition data, 2706 for play, 4312 for greeting, and 2629 for social licking data. The total number of data-points was 10,999. So that an assessment of cohesion and relationships between the adult pride members could be carried out, analyses were also conducted on the pride excluding any data from cubs. This resulted in 70 data-points for play, 1510 for greeting, 1043 for social licking and 1350 for group composition (total 3973 data-points).

Group composition and social interaction behaviour was collected each time the research vehicle entered the site. Upon entering the site, telemetry was used to locate each lion. Each lion was fitted with a radio collar as part of ALERT’s regular monitoring of this pride, and were therefore in place prior to the start of the current study. For group composition data, a 50 m threshold was imposed and observations were recorded of which individual lions were present in the group and which were not. If the grouping changed during observation, this was documented and once the group had settled a note was made of which members of the pride were now present and which were absent.

Social interactions were recorded continuously to provide a true measure of frequency throughout the data collection period. Behaviours identified for the purposes of the current study were greeting with head rubbing, social licking, and play. When any of these occurred, a record was made of the time of interaction, which lion initiated it, which lion was the object of it, which behaviour had occurred (play, greeting or social licking), and whether the interaction was accepted peacefully by the recipient. Definitions of each of these behaviours were consistent with those provided by Schaller [24]. Greeting by head-rubbing was noted if one lion approached another from the front, behind or side, and rubbed another touching its cheeks in passing. Social licking was recorded if one lion licked the head, upper neck, shoulder and chest, back and side, abdomen or any other part of another lion’s body. Instances of social play were classified and recorded if the activity involved two or more lions and fell into one of Schaller’s four categories: chasing, wrestling, pawing, stalking and rushing. The display of these behaviours must appear to have no survival value (e.g. occur in the absence of hunting or eating) for them to be defined as play. To ensure independence of data as much as possible, a separate event was deemed to occur if there was a gap of at least 1 minute, with no further display of the behaviour, between the finish of one bout and the start of another.

### Data Analysis

All of the social data in this analysis (play, greeting, social grooming and all social combined) was compiled into asymmetric (directional) matrices based on data type. Group composition data was evaluated and compiled into symmetric (undirectional) matrices based on the simple ratio index: 
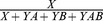
 where X is the number of times individuals *A* and *B* are seen in the same group, Y_A_ is the number of times individual *A* is seen without individual *B*, Y_B_ is the number of times individual *b* is seen without individual *A*, and Y_AB_ is the number of times that individuals *A* and *B* are seen in different groups. This analysis used a modified simple ratio index where Y_AB_ was excluded as it cannot be determined with sufficient confidence. These are co-occurrence networks as they overlap in the same time-frame. The matrices were then analysed in the social network analysis programme UCINET [38], which was used to calculate the values for density, transitivity, clique groups, degree (indegree, outdegree), and betweenness for each data type.

The density of a matrix is a calculation of the number of dyadic associations (edges) present in a network as a proportion of all possible connections. High values represent highly connected networks. A value of 1 represents a fully connected network (complete graph) and a value of 0 marks a fully unconnected one (empty graph). Transitivity provides a measure of triadic associations of a given matrix such that if there are ties (edges) connecting A and B, and ties connecting B and C, transitivity compares the connection between A and C to these associations. If the connection between A and C is stronger than the ties between A and B, and B and C, this is considered strong transitivity. The higher the transitivity value the greater the likelihood that there is a strong triadic relationship between A and C. Directionality is taken into effect within UCINET in assessing transitivity between triads. Transitivity is calculated using the formula: 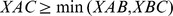. UCINET was also used to evaluate individuals within the group using degree for symmetric matrices, indegree and outdegree for asymmetric matrices, and betweenness. The degree defines the number of interactions each individual has with other individuals within a network. More specifically, for directional networks where an individual both gives and receives social interactions (i.e. social grooming), indegree describes those interactions an individual receives and outdegree refers to the interactions instigated by the target individual. Spearman’s correlation coefficient was calculated on indegree and outdegree within and between the networks.

The betweenness of a node is a measure of an individual’s centrality within the group. It measures the number of shortest paths individuals must go through in order to connect to a target individual. Betweenness is calculated using the following formula: 
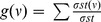
 where *σ_st_* is the total number of shortest paths from node *s* to node *t* and *σ_st_(v)* is the number of those paths that pass through *v*. More central individuals are represented by a high value as they connect other members of the group who may not already be connected, and may also serve as a connecting bridge between subgroups. Although the pride under investigation is small, a measure of betweenness indicates differences in preferential interactions of individuals. Kendall’s tau correlation was used to examine any relationships for betweenness (centrality) and degree (indegree and outdegree). This facilitated an analysis of any association between social influence and social power.

Data values for degree, indegree, outdegree and betweenness were normalized in UCINET. The normalization of these values allows for a comparison of data across different datasets such that the effects of sample size and other influences are eliminated from the comparison.

The social network visualization tool NETDRAW was used to develop sociograms from the original social network and group composition network matrices and clique network diagrams from the UCINET clique analysis results. SOCPROG [39] was used to analyse the significance of the relationship between the social and group composition matrices to each other and against a random network (generated in UCINET) using the Mantel Test [40]. The Mantel Test is a permutation test that evaluates significance of two matrices with the same individuals using the null hypothesis that there is no relationship between the two matrices. The social matrices (play, social grooming and greeting), as well as the group composition data were compared to one another, a random network and association matrices based on the age, gender and kinship of the individuals in the social groups.

## Results

Analyses of the structural patterns at group (density, transitivity, clique) and individual (social interactions) level were conducted for the Ngamo pride, including and excluding the cubs (AS4, AS5, KE3, KE4, AT1). It is acknowledged that any differences found according to cub inclusion may represent a biological fact or group size.

Measures of density and transitivity show all networks for the Ngamo pride are highly connected and each individual’s associates are associated, when the data includes cubs (Table 2), and when cubs are removed from the analysis (Table 3). The pride is fully connected for group composition, indicating strong spatial cohesion. A completely connected network is one where all nodes interact with all other nodes in both directions, whereas a network with low connectivity has very few nodes connected to one another. In the current case, the network is highly connected as it is not complete, but most nodes interact with most other nodes.

**Table 2:** Density and Transitivity of all observed networks, including cub data.

	Play	Greeting	SocialLicking	AllSocial	Composition
Density	0.894	0.977	0.902	0.992	1
Transitivity (Strong)	70.909	77.727	71.364	70.227	67.273

**Table 3:** Density and Transitivity for all observed networks, excluding cub data.

	Play	Greeting	SocialLicking	AllSocial	Composition
Density	0.667	0.976	0.81	0.976	1
Transitivity(Strong)	86.667	73.81	72.857	69.048	66.667

**Figure 1:**
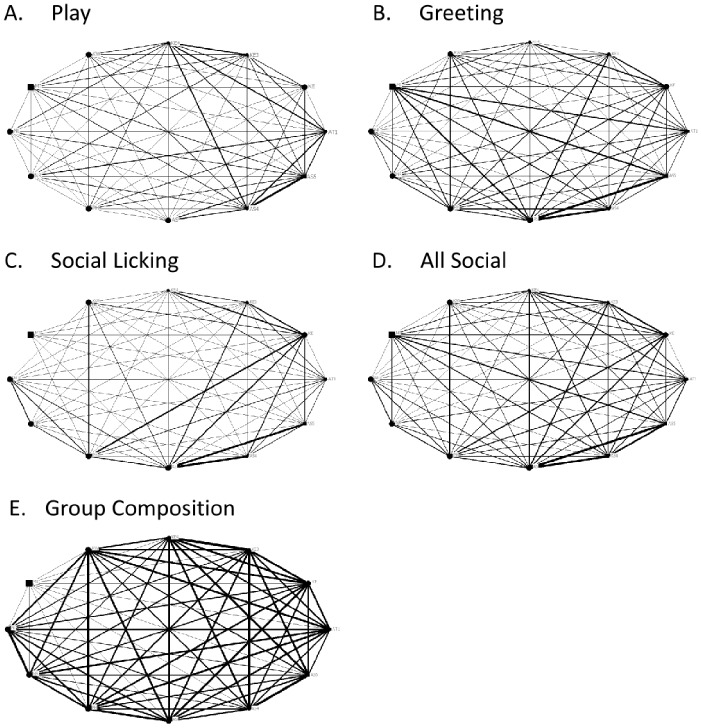
Sociograms for all networks in the Ngamo pride (including cubs). Sociograms illustrating the networks for A) play, B) greeting, C) social licking, D) all social interactions combined, and E) group composition for the Ngamo pride when cubs are included in the dataset and analysis. Line thickness represents the strength of the association between dyads. Circles represent female lions and squares represent male lions. The size of the circle or square is directly proportional to the age of the individual; larger shapes denote older individuals.

**Figure 2:**
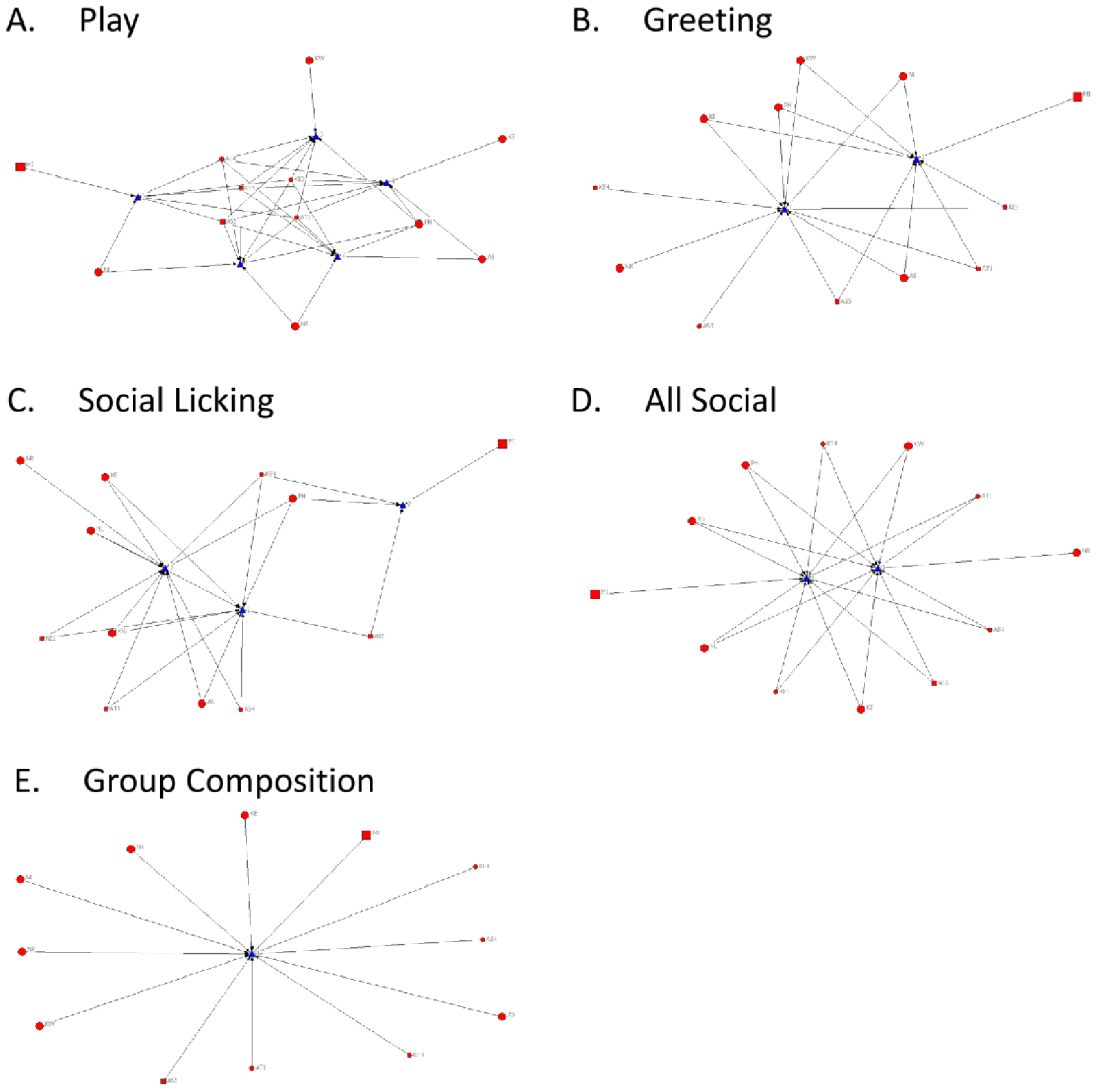
Clique analysis for Ngamo pride (including cubs). Clique analysis illustrating the sub-groups present in the networks for A) play, B) greeting, C) social licking, D) all social interactions combined, and E) group composition. Circles represent individual lions and triangles indicate the cliques they are involved in.

**Figure 3:**
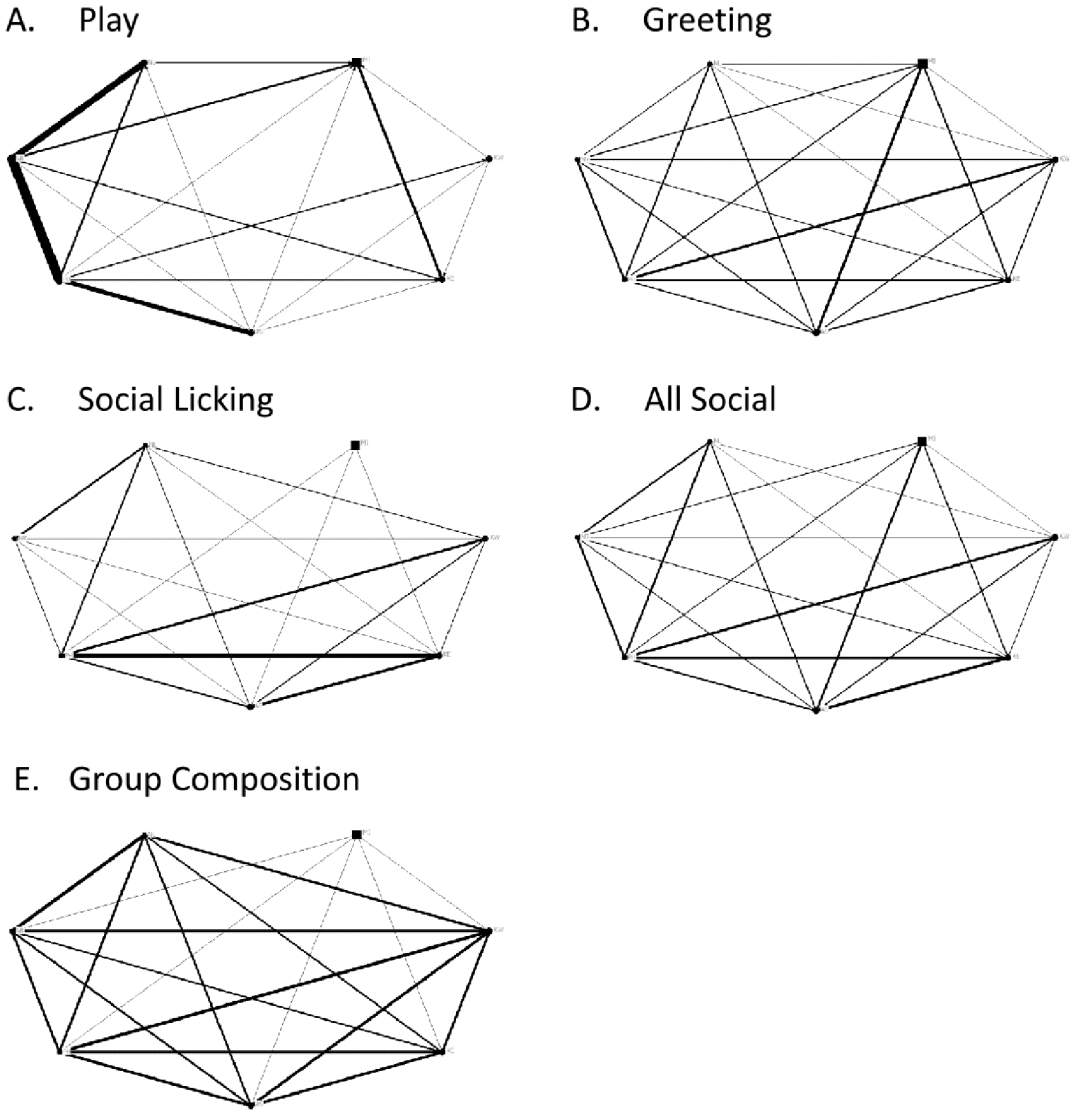
Sociograms for all networks in the Ngamo pride (excluding cubs). Sociograms illustrating the A) play, B) greeting, C) social licking, D) all social interactions combined, and E) group composition for the Ngamo pride when cubs are excluded from the dataset and analysis. Line thickness represents the strength of the association between dyads.

**Figure 4:**
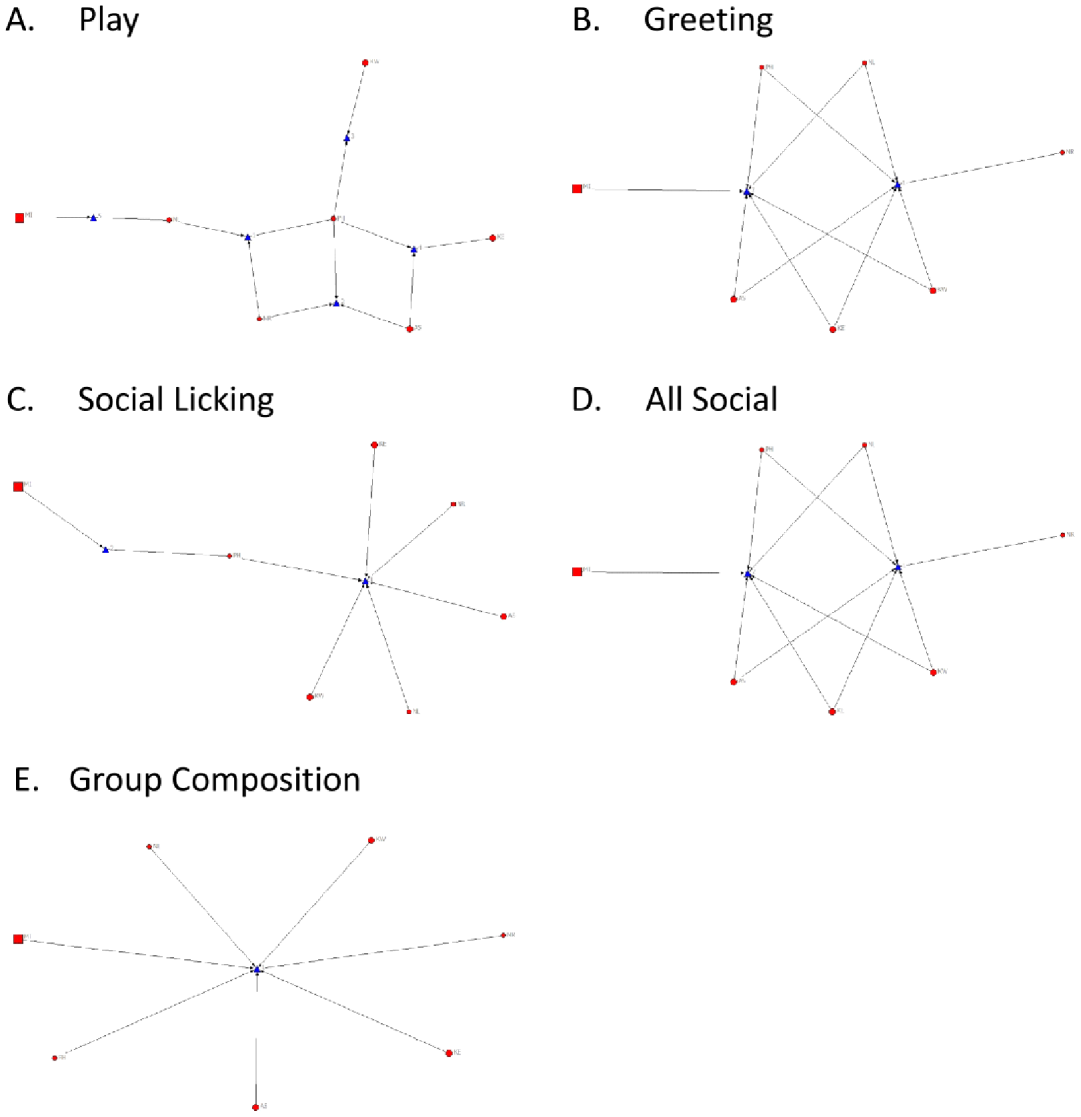
Clique analysis for all networks in the Ngamo pride (excluding cubs). Clique analysis illustrating the sub-groups present in the networks for A) play, B) greeting, C) social licking, D) all social interactions combined, and E) group composition. Circles represent individual lions and triangles indicate the cliques they are involved in.

The matrices for all ties (sociograms) and cliques in the Ngamo pride are visualised in Figures 1 and 2 (including cubs) and Figures 3 and 4 (excluding cubs). The thicker the line between individuals, the more interactions were observed between them. High transitivity is observed in all networks indicating overall cohesion, but cliques are evident. The transitivity values observed are close to the maximum that can be calculated within UCINET. A clique describes a sub-group of individuals within the network, who share closer interactions with one another than with other members of the network. The central position of individual lions within each network is calculated as betweenness, including (Table 4) and excluding (Table 5) cubs.

**Table 4:** Betweenness (centrality) values for the Ngamo pride, including cub data.

	Play	Greeting	SocialLicking	AllSocial
AS	0.345	0.341	0.642	0.091
AS4	2.059	0.000	0.101	0.091
AS5	2.059	0.341	2.814	0.091
AT1	2.059	0.341	0.642	0.091
KE	0.000	0.341	0.642	0.091
KE3	2.059	0.341	0.642	0.091
KE4	2.059	0.000	3.066	0.091
KW	0.000	0.341	0.101	0.091
MI	0.215	0.000	0.000	0.000
NL	0.303	0.341	0.101	0.091
NR	0.361	0.000	0.000	0.000
PH	1.21	0.341	3.066	0.091

**Table 5:** Betweenness (centrality) values for the Ngamo pride, excluding cub data.

	Play	Greeting	Social Licking	All Social
AS	3.611	0.667	3.333	0.667
KE	0.000	0.667	3.333	0.667
KW	0.000	0.667	0.000	0.667
MI	1.944	0.000	0.000	0.000
NL	16.667	0.667	0.000	0.667
NR	7.500	0.000	0.000	0.000
PH	26.944	0.667	20.00	0.667

For the group composition network, UCINET did not identify any cliques (Figs. 2 & 4, E), although the pride male MI is more likely to apart than other lions (Figs. 1 & 3, E). Betweenness for group composition is 0 for all lions, indicating no centrality for this network.

The play network contains 5 cliques (Figs. 2 & Fig. 4, A). The least playful lions are MI, KW and KE who are only involved in one clique. The most centrally connected lions for the play network are the cubs (AS4, AS5, AT1, KE3, KE4) and PH (Table 4). KE (mother to KE3 and KE4) and KW have betweenness values of 0 indicating they are not a point of social connection in the play network.

The greeting network has 2 cliques (Figs. 2 & 4, B). Two cubs, KE4 and AS4, are the least connected in the greeting network along with MI and NR (Figs. 2 & 4, B). Centrality for the greeting network is evenly distributed between lions, except for MI, NR, KE4 and AS4, who only contribute to one clique (Fig. 2, B).

The subgroupings within the social licking network are reduced from 3 cliques to 2 cliques when cubs are removed from the dataset and analysis (Fig. 2 & 4, C). When cubs are removed from the analysis all adult lions constitute one clique, except for MI who is involved in a separate second clique with PH. There are no observed social licking interactions between MI, and the three spayed females (KW, NL and NR). The most central adult lion for the social licking network is PH (Table 5) and the most socially connected cubs are KE4 and AS5. MI and NR are not connected at all, and NL, KW and AS4 display weak centrality.

When all social interactions are combined there are two cliques evident within the pride with all lions, except for MI and NR, involved in both (Figs. 2 & 4, D). Centrality is evenly distributed across the pride with the exception of MI and NR who exhibit no betweenness for any social network.

The number of social interactions each lion received (indegree) and initiated (outdegree) for play, greeting, social licking, and all social combined, are presented in Table 6 (with cubs included) and Table 7 (cubs excluded).

**Table 6:** Degree values for the Ngamo pride, including cub data.

	Indegree				Outdegree			
	Play	Greeting	Licking	All Social	Play	Greeting	Licking	All Social
AS	7.995	36.56	21.459	40.445	2.273	14.679	35.25	27.836
AS4	17.589	3.817	18.306	21.081	31.719	23.416	7.05	38.024
AS5	19.368	3.778	17.625	21.756	22.678	24.518	7.483	33.859
AT1	14.97	10.783	10.823	21.165	19.615	16.962	10.39	28.033
KE	7.658	21.448	18.306	28.033	4.298	13.892	29.87	25.978
KE3	17.836	4.132	14.224	19.589	18.874	18.418	10.451	28.68
KE4	20.109	3.857	11.936	19.645	22.184	15.191	8.967	27.582
KW	3.953	13.459	11.503	17.112	3.113	10.901	6.37	12.468
MI	7.362	28.729	0.433	24.937	0.593	0.984	2.103	1.998
NL	4.792	7.871	8.472	12.215	2.372	9.13	14.657	14.551
NR	5.83	8.776	7.297	12.919	3.211	13.577	9.709	15.958
PH	6.275	26.486	22.202	32.62	2.767	8.028	20.284	16.549

**Table 7:** Degree values for the Ngamo pride, excluding cub data.

	Indegree				Outdegree			
	Play	Greeting	Licking	All Social	Play	Greeting	Licking	All Social
AS	12.500	36.400	33.495	47.661	6.944	40.133	27.832	46.589
KE	6.944	22.133	33.333	36.745	9.722	37.467	35.761	49.610
KW	1.389	19.867	25.081	29.727	8.333	32.000	14.078	32.456
MI	16.667	43.600	0.162	33.138	2.778	2.533	2.751	3.704
NL	18.056	13.067	17.799	21.54	9.722	27.467	26.699	36.482
NR	13.889	14.667	15.049	20.76	36.111	39.333	19.417	42.982
PH	27.778	51.600	43.851	66.082	23.611	22.400	42.233	43.470

When cubs are included in the analysis, they are the highest receivers and initiators of play interactions. The highest adult receivers for play are the parents of the cubs AS, KE and MI (except AT1, whose mother is not present in the pride) (Table 6). When cubs are excluded, PH receives the most play interactions, and initiates the second highest (after NR) (Table 7). Strong associations are evident between PH and NR, and NR and NL for play (Fig. 3A). KE, KW and NR all initiate more play interactions than they receive (Table 7), and KW is the lion least likely to receive a play interaction. MI is the lion least likely to initiate play. Spearman’s correlation coefficient shows play indegree and outdegree are positively correlated (*r_s_* = 0.699, *p* = 0.001). When cubs are included, lions who receive the most play interactions (cubs) are those who initiate the most social interactions overall (*r_s_* = 0.804, *p* = 0.002), receive the fewest greetings (*r_s_* = −0.825, *p* = 0.001), initiate the most greetings (*r_s_* = 0.853, *p* = 0.00), and receive the most social interactions overall (*r_s_* = 0.776, *p* = 0.003). Kendall’s tau on pride centrality for play (play betweenness) including cub data, shows this network to be positively associated with play indegree (*τ* = 0.614, *p* = 0.008), play outdegree (*τ* = 0.548, *p* = 0.018), greeting outdegree (*τ* = 0.548, *p* = 0.018), all social outdegree (*τ* = 0.647, *p* = 0.005), and negatively correlated with greeting indegree (*τ* = −0.481, *p* = 0.038). So those lions central to the play network (cubs), initiate and receive the most play interactions, initiate the most greeting and social interactions overall, but receive the fewest greetings. This is consistent with cub status in a pride and they exert a heavy influence on this network. When cubs are removed from the analysis, only play betweenness and play indegree are correlated. PH is the most central adult to the play network and receives the most play interactions (Table 7). Mantel tests for correlations of the play matrix and attribute characteristics, show positive associations for half-siblings (*r = *0.5047, *p* = 0.0008), full-siblings (*r = *0.3856, *p* = 0.0021), and age (*r = *0.1633, *p* = 0.0341), when cubs are included (Table 8). This is an expected result as related cubs of similar age are central to the play network. When cubs are removed from the analysis, the play network is associated with the greeting network (*r* = 0.3102, *p* = 0.0284) (Table 9).

**Table 8:** Mantel Tests for all networks and attribute characteristics, including cub data.

	Play	Greeting	Licking	All Social	Composition
Play	/	0.1551	0.5267	0.0001	0.0043
Greeting	/	/	0.0955	0.0000	0.2368
Licking	/	/	/	0.0000	0.0000
All Social	/	/	/	/	0.0001
Gender	0.5688	0.4077	0.0977	0.2704	0.0433
Half Sib	0.0008	0.7081	0.0100	0.0019	0.0007
Full Sib	0.0021	0.6642	0.1367	0.0215	0.0002
Age	0.0341	0.8427	0.2435	0.2131	0.0396
Random	0.6149	0.1044	0.3871	0.2261	0.2389
**Matrix Coefficient**					
Play	/	0.0918	−0.0263	0.5554	0.3435
Greeting	/	/	0.1201	0.7395	0.0743
Licking	/	/	/	0.5287	0.4526
All Social	/	/	/	/	0.433
Gender	−0.0605	0.0414	0.2344	0.1047	0.516
Half Sib	0.5047	−0.0415	0.2779	0.36	0.52
Full Sib	0.3856	−0.0319	0.115	0.2296	0.4053
Age	0.1633	−0.0606	0.0679	0.0762	0.2154
Random	−0.0277	0.106	0.0266	0.0658	0.0708

**Table 9:** Mantel Tests for all networks and attribute characteristics, excluding cub data.

		Play	Greet	Licking		All Social	Composition		
Play		/	0.0284	0.1805		0.03	0.2022		
Greeting		/	/	0.0262		0.0002	0.0428		
Licking		/	/	/		0.0005	0.0042		
All Social		/	/	/		/	0.0016		
Gender		0.5775	0.2897	0.144		0.1434	0.0072		
Half Sib		0.3459	0.0008	0.0094		0.0076	0.0087		
Full Sib		0.2086	0.4659	0.1808		0.2015	0.0576		
Random		0.2529	0.2893	0.5103		0.3739	0.7732		
**Matrix Coefficient**									
Play	/		0.3102		0.1828	0.356		0.2056	
Greeting	/		/		0.2655	0.7965		0.2433	
Licking	/		/		/	0.7926		0.6722	
All Social	/		/		/	/		0.5769	
Gender	0.1325		0.1582		0.5154	0.4236		0.9261	
Half Sib	0.0776		0.2656		0.782	0.6521		0.6667	
Full Sib	0.1472		0.0148		0.1832	0.1302		0.3283	
Random	0.1119		0.0905		−0.0008	0.061		−0.1208	

Parents of the cubs, AS, KE and MI, have high greeting indegree (Table 6). An exception to this is PH who receives a higher number of greetings than KE. The youngest cubs (AS4, AS5, KE3, KE4) receive the fewest greetings, but the older AT1 receives more than adults NL and NR. As receiving greetings may signal social power and dominance within a pride, these values may reflect PH’s high ranking and the low social status of the young cubs and full-sisters NL and NR. Further illustration of these positions may be gleaned from greeting outdegree values where the highest initiators are the cubs, their mothers and NR, and the lowest initiators are MI and PH. When cubs are removed from the analysis, PH receives the most greetings (followed by MI), and initiates the fewest (after MI) (Table 7). Only MI and PH receive more greetings than they receive. Greeting indegree is only negatively correlated with greeting outdegree (*r_s_ = *−*0.699, p = 0.01)* when cubs are included in the analysis. When cubs are removed from the analysis, greeting indegree is negatively correlated with all social interactions outdegree (*r_s_* = −0.699, *p* = 0.01); however, this is skewed with the presence of MI in the data who initiates very few social interactions overall. When MI is removed from the analysis, the correlation is non-significant (*r_s_* = −0.509, *p* = 0.110). Mantel test analysis shows the greeting network is associated with half-siblings (*r* = 0.2656, *p* = 0.0008) (Table 9).

PH and AS receive the most social licking (Table 6 & 7). MI receives the fewest social licking interactions, with NL and NR exhibiting the lowest indegree values amongst the adult females. Social licking is most likely to be initiated by AS, KE, PH and NL. When cubs are excluded, PH is the individual initiating most social licking in the pride (Table 7). MI is the least likely to initiate any social licking behaviour. Social licking indegree and outdegree are correlated when cubs are removed from the data (*r_s_* = 0.857, *p* = 0.014). Social licking betweenness is negatively correlated with social licking indegree (*τ* = −0.530, *p* = 0.023) when cubs are included in the data, but are positively correlated (*τ* = 0.0816, *p* = 0.017) when excluded. Centrality to the social licking network also correlates with social licking outdegree (*τ* = 0.816, *p* = 0.017) when cubs are excluded. Mantel tests show social licking to be associated with half-siblings when cubs are included (*r* = 0.2779, *p* = 0.0100) (Table 8) and excluded (*r* = 0.7820, *p* = 0.0094) (Table 9). The social licking network is also associated with the greeting network (*r* = 0.2655, *p* = 0.0262) (Table 9).

Across all social interactions, PH and AS receive the most and NL and NR the least. When cubs are included, they are the highest initiators of social behaviours across all networks. When excluded, the highest initiators are KE, AS, PH and NR. The pride are highly involved as receivers and initiators of social interactions, with the exception of MI who initiates very few.

Group composition is associated with play (*r* = 0.3435, *p* = 0.0043), all social interactions (*r* = 0.4330, *p* = 0.0001), gender (*r* = 0.5160, *p* = 0.0433), age (*r* = 0.2154, *p* = 0.0396), half (*r* = 0.5200, *p* = 0.0007) and full (*r* = 0.4053, *p* = 0.0002) siblings, when cubs are present in the data (Table 8). When cubs are removed, group composition is related to greeting (*r* = 0.2433, *p* = 0.0458), social licking (*r* = 0.6722, *p* = 0.0042), all social (*r* = 0.5769, *p* = 0.0016) networks, and the attributes of gender (*r* = 0.9261, *p* = 0.0072), half (*r* = 0.6667, *p* = 0.0087) and full-siblings (*r* = 0.3283, *p* = 0.0576) (Table 9). Age was removed from these Mantel tests as all adult are similar age. As group composition has a density of 1, these results are expected. The null hypothesis that associations in each of the networks were random was rejected.

## Discussion

This study was an exploration of cohesion and relationships within a constructed pilot captive-bred lion pride whose cubs are intended for wild-release. A social network analysis of the dataset indicates that the pride is a highly cohesive group, both when cubs are included and excluded from the assessment. As a group, the pride is highly connected (density) with associates associated (transitivity) across all observed networks.

Analyses show associations in each network are not random, but individual lions exhibit preferences. Consequently, some lions are more central to networks than others. Social network analysts emphasise the importance of central, or ‘keystone’ individuals, who are the social glue ensuring cohesion within group-living species. In the current study PH is the keystone individual for this pride. She is central to the play and social licking networks, and is involved in all but one clique across every network. Consequently she connects peripheral individuals, as well as the dominant pride male, MI, in networks. As well as socially influential, PH is arguably socially powerful if we consider greeting to be an indicator of hierarchy [24].

Genetically unrelated to the rest of the pride, NL and NR’s position is more tenuous. They do not have high centrality to any network and have very weak connections with MI. This might be explained in part by their sterility as well as kinship. KW (also spayed) also has poor associations with MI, and is not a social connection point in the play and social licking networks. NL is not a point of social connection for social licking, and her sister NR has no centrality for social licking, greeting, and all social combined. NR contributes to just one clique for these networks. NR and NL’s strongest associations are with each other. NR is a playful lion with the cubs AT1, KE3 and AS4, and NL is playful with her sister and PH. In terms of social interactions, both NL and NR are either uninvolved or initiate more than they receive. The Mantel tests show that kinship is associated with the networks, which may also explain why NL and NR find themselves on the edges of the pride. In a pride comprising of related and unrelated lions, genetics are linked to network position. Individuals seem to prefer to socially interact with their kin.

AT1’s mother was removed from the pride when AT1 was 9 months old. Her social position is therefore interesting in terms of how well she’s integrated into the pride and her relationships with other lions. Visual representations of the networks show AT1 to be well-integrated. She’s involved in most cliques and seems to be connected to other individuals. Her behaviour, in particular the display of playing, is consistent with being a cub.

As expected, AS and KE have strong associations with their cubs. AS receives more interactions than she initiates (even when cubs are removed from the analysis), and has some centrality in all networks. KE, initiates more social interactions than she receives (when cubs are removed from the analysis), and is not a point of social connection in the play network.

Previous research suggests an indicator of social cohesion is spatial proximity. The group composition data shows the Ngamo pride are in close proximity to one another across the networks. The exception is the pride male, MI. However, spatial cohesion may be conflated with social cohesion [35] owing to the size of the site and the fence upon this behaviour. The physical constraint on space may facilitate individuals to share space.

There are other reasons to be cautious with these results. Hunting and reproductive behaviour are not accounted for, and neither are nocturnal activities. A further criticism of social network analysis is that of temporality. Data across a period of time is analysed and presented as a snapshot of the pride. The current study concerns a relatively short period of time, but there will inevitably be variation within the pride over this time-frame which are not evident in the analysis. Also, not included in the current study is consideration of the dispositions of the lions themselves. There may be reasons why some adult lions are dispositionally minded to engage in some behaviour and not others. PH is a keystone lion, but social network analysis provides a study of ‘how’ rather than ‘why’. Finally, the sample size is extremely small. This pilot study concerns a single pride of 12 lions. However, the intention is not to provide a generalizable account of lion behaviour per se, but to illustrate structural and individual association processes involved in pride cohesion, and establish whether this has occurred at this stage. Fulfilling the criteria advances the progress of these captive-bred lions to a larger managed ecosystem, comprising of competitive species. This study also flags the importance of keystone individuals in maintaining group cohesion, especially when kinship ties are absent.

Measures of association are important for ex situ reintroduction programmes in order to assess cohesion within a pride and the most central individuals. An assessment of pride structure and the associations between individuals is fundamentally important to conservation strategies which split wild prides for reintroduction purposes into other areas. Whilst network links can be reorganized prior to being split previous attempts to reintroduce from wild sources have resulted in the fragmentation of groups as individuals have not been sufficiently cohesive prior to release [2], [3]. Moreover, published studies do not exist of the impact such practices have upon the remaining pride once some individuals have been removed. The reorganization of networks post-split needs some empirical attention. Careful analysis is required to guide decisions over which individuals should be translocated pre-split. The ALERT pride contains related and unrelated females. Where such practices may become increasingly common with the rise of ex situ conservation practices, identification of keystone individuals will be vital in ensuring released groups do not fragment. Further, it would be beneficial to have studies of cohesion in wild prides such that ex situ attempts could be assessed against naturally occurring groups. Whilst the current study does not claim to provide an account of social cohesion within lion prides per se, it does offer a first exploration into the relationships that exist within a particular group containing related and unrelated individuals.

## References

[1] Killian PJ, Bothma J du P (2003) Notes on the social dynamics and behaviour of reintroduced lions in the Welgevonden Private Game Reserve. South African Journal of Wildlife Game Reserve 33(2): 119–124.

[2] Trinkel M, Ferguson N, Reid A, Reid C, Somers M (2008) Translocating lions into an inbred lion population in the Hluhluwe-iMfolozi Park, South Africa. Animal Conservation 11: 138–143.

[3] Riggio J, Jacobson A, Dollar L, Bauer H, Becker M, et al. (2012) The size of savannah: A lion’s (Panthera leo) view. Biodiversity Conservation: DOI 10.1007/s10531-012-0381-4.

[4] Bauer H, Nowell K, Packer C (2008) Panthera leo. In: IUCN Red List of Threatened Species. Switzerland: Gland. Available: http://www.iucnredlist.org. Accessed 31 July 2013.

[5] Abell J, Kokeŝ R, Youldon D (2013a) The long-term viability of current lion conservation strategies: A role for ex situ reintroduction. Open Science Repository Natural Resources and Conservation Online: doi:10.7392/openaccess.70081975.

[6] Abell J, Kokeŝ R, Youldon D (2013b) A framework for the ex situ reintroduction of the African lion (*Panthera leo*). Open Science Repository Natural Resources and Conservation Online: doi: 10.7392/openaccess.70081986.

[7] Beck BB, Rapaport LG, Stanley-Price MR, Wilson AC (1994) Reintroduction of captive-born animals. In: Olney PJS, Mace GA, Feiste ATC, editors. Creative Conservation: Interactive Management of Wild and Captive Animals. London: Chapman & Hall. PP. 265–286.

[8] Fischer J, Lindenmayer DB (2000) An assessment of the published results of animal relocations. Biological Conservation 96: 1–11.

[9] Jule KR, Leaver LA, Lea SEG (2008) The effects of captive experience on reintroduction survival in carnivores: A review and analysis. Biological Conservation 14: 355–363.

[10] Kleiman DG (1989) Reintroduction of captive mammals for conservation. BioScience 39: 152–161.

[11] Schulte BA (2000) Social structure and helping behaviour in captive elephants. Zoo Biology 19: 447–459.

[12] Carlstead K (1996) Effects of captivity on behaviour of wild mammals. In: Kleiman DG, Allen M, Thompson K, Lumpkin S, Harris H, editors. Wild Mammals in Captivity: Principles and Techniques. Chicago: University of Chicago Press. 317–333.

[13] McDougall PT, Réale D, Sol D, Reader SM (2006) Wildlife conservation and animal. temperament: Causes and consequences of evolutionary change for captive, reintroduced, and wild populations. Animal Conservation 9: 39–48.

[14] Adamson J (2000) Born Free: The Full Story. New York: Pantheon. PP. 432.

[15] Patterson G (1994) Last of the Free. New York: St Martin’s Press. PP. 157.

[16] Bertram B (1978) Pride of Lions. New York: Charles Scribner’s Sons. PP. 253.

[17] Aureli F, Schaffner CM, Boesch C, Bearder SK, Call J, et al (2008) Fission-fusion dynamics: New research frameworks. Current Anthropology 49(4): 627–654.

[18] Packer C, Pusey AE (1997) Divided we fall: Cooperation among lions. Scientific American. May: 32–29.

[19] Mosser A, Packer C (2009) Group territoriality and the benefits of sociality in the African lion, Panthera leo. Animal Behaviour 78: 359–370.

[20] Packer C, Pusey AE, Ebley LE (2010) Egalitarianism in female African lions. Science 293: 690–693. 10.1126/science.1062320 11474110

[21] Spong G, Creel S (2004) Effects of kinship on territorial conflicts among groups of lions, Panthera leo. Behavioral Ecology and Sociobiology 55(4): 325–331.

[22] Hamilton WD (1964) The genetical evolution of social behaviour. Journal of Theoretical Biology 7(1): 1–52. 5875341 10.1016/0022-5193(64)90038-4

[23] Blaustein AR, Bekoff M, Daniels TJ (1987) Kin recognition in vertebrates. (excluding primates): Empirical evidence. In D.J.C. Fletcher and C.D. Mitchener (eds) Kin Recognition in Animals. Chichester: Wiley & Sons. PP. 287–332.

[24] Schaller GB (1972) The Serengeti Lion. Chicago: The University of Chicago Press. PP. 480.

[25] Lancy DF (1980) Play in species adaptation. Annual Review of Anthropology IX: 471–495.

[26] Pellis SM, Pellis VC (1996) On knowing it’s only play: The role of play signals in play fighting. Aggression and Violent Behavior 1(3): 249–268.

[27] Schenkel R (1966) Play, exploration and territoriality in the wild lion. Symposium of the Zoological Society of London 18: 11–22.

[28] Smith PK (1982) Does play matter? Functional and evolutionary aspects of animal and human play. Behavioral and Brain Sciences 5: 139–184.

[29] Hellaye YL, Benoît G, Jamart A, Curtis DJ (2010) Acquisition of fission-fusion social organization in a chimpanzee (*Pan troglodytes troglodytes*) community released into the wild. Behavioural Ecology and Sociobiology 64: 349–360.

[30] Smith JE, Powning KS, Dawes SE, Estrada JR, Hopper AL, et al (2011) Greetings promote cooperation and reinforce social bonds among spotted hyaenas. Animal Behaviour 81: 401–415.

[31] Packer C (1986) The ecology of sociality in felids. Ecological Aspects of Social Evolution: 429–451.

[32] Krause J, Croft DP, James R (2007) Social network theory in the behavioural sciences: potential applications. Behavioral Ecology and Sociobiology 62: 15–27. 10.1007/s00265-007-0445-8 PMC7079911 32214613

[33] Sih A, Hanser SF, McHugh KA (2009) Social network theory: New insights and issues for behavioural ecologists. Behavioral Ecology and Sociobiology 63: 975–988.

[34] Krause J, Lusseau D, James R (2009) Animal social networks: An introduction. Behavioral Ecology and Sociobiology 63: 967–973.

[35] Wey T, Blumstein DT, Shen W, Jordán F (2008) Social network analysis of animal behaviour: A promising tool for the study of sociality. Animal Behaviour 75: 333–344.

[36] Wilson EO (1975) Sociobiology: The New Synthesis. Cambridge MA: Harvard University Press. PP. 697.

[37] Law G, Macdonald A, Reid A (1997) Dispelling some common misconceptions about the keeping of felids in captivity. International Zoo Yearbook 35: 197–205.

[38] Borgatti SP, Everett MG, Freeman LC (2002) Ucinet for Windows: Software for Social Network Analysis. Harvard MA: Analytic Technologies. Available: https://sites.google.com/site/ucinetsoftware/news/newbookonsna. Accessed 31 July 2013.

[39] Whitehead H (2008) Analyzing Animal Societies: Quantitative Methods for Vertebrate Social Analysis. Chicago: University of Chicago Press. pp. 336.

[40] Mantel NA (1967) The detection of disease clustering and a generalized regression approach. Cancer Research 27: 209–220. 6018555

